# Patient with Phenylketonuria and Intellectual Disability—Problem Not Always Caused Exclusively by Insufficient Metabolic Control (Coexistence of PKU and Alazami Syndrome)

**DOI:** 10.3390/ijerph19052574

**Published:** 2022-02-24

**Authors:** Michał Patalan, Alicja Leśniak, Krzysztof Bernatowicz, Hanna Romanowska, Elżbieta Krzywińska-Zdeb, Mieczysław Walczak, Maria Giżewska

**Affiliations:** 1 Department of Pediatrics, Endocrinology, Diabetology, Metabolic Diseases and Cardiology, Pomeranian Medical University in Szczecin, Unii Lubelskiej 1, 71-252 Szczecin, Poland; alicjalesniak60@gmail.com (A.L.); hanna.romanowska33@gmail.com (H.R.); zdebela@op.pl (E.K.-Z.); ghmwal@pum.edu.pl (M.W.); maria.gizewska@gmail.com (M.G.); 2 Independent Public Clinical Hospital No. 1, Pomeranian Medical University in Szczecin, Unii Lubelskiej 1, 71-252 Szczecin, Poland; 3 Adult Genetics Unit, Royal Adelaide Hospital, Port Road, Adelaide 5000, Australia; ka.bernatowicz@gmail.com

**Keywords:** Alazami syndrome, phenylketonuria, PKU, WES

## Abstract

The authors present a case report of a boy with a classical form of phenylketonuria and Alazami syndrome. The inborn error of phenylalanine metabolism was diagnosed in the neonatal period based on population new-born screening. Despite early implementation of a low-phenylalanine diet and good biochemical control, the patient developed behavioural disorders and intellectual disability. He also presented with dysmorphic features. After long and extensive attempts to establish the genetic cause of this unusual phenotype, finally, at the age of 16 the boy was diagnosed with Alazami syndrome based on whole exome sequencing. The authors discussed the problem of neuropsychological disorders in patients with phenylketonuria and described typical clinical symptoms of Alazami syndrome. It was emphasized that the presence of one monogenic disease does not exclude the coexistence of another one.

## 1. Introduction

Phenylketonuria (PKU) [MIM #261600] is the most frequent inborn error of amino acid metabolism and is related to the disruption of the phenylalanine (Phe) metabolic pathway. In Poland, the prevalence of PKU is 1 case per 8300 live births [1]. The pathophysiology of the disorder is associated with complete or partial deficiency of a liver-expressed enzyme phenylalanine hydroxylase (PAH), which is responsible for hydroxylation of Phe to tyrosine (Tyr).

In untreated patients with PKU or those with poor metabolic control, Phe accumulating in the blood and body fluids crosses the blood–brain barrier leading to multidirectional damage of the central nervous system (CNS). Such patients consequently present intellectual disability and other neuropsychological deficits [2]. Excessive concentration of Phe interferes with the functioning of myelin-producing oligodendrocyte glial cells, leading to impairment in myelin synthesis or demyelination. This results in a decrease in conduction velocity of action potentials and disrupts the formation of both neuronal connectivity and white matter integrity. Hyperphenylalaninemia (HPA) also inhibits the transport of Tyr and tryptophan (Trp) through the blood–brain barrier, inducing deficiency of these amino acids in CNS. Additionally, limited activity of the PAH enzyme and disruption of proper Phe to Tyr conversion is another factor causing Tyr deficiency. Both Tyr and Trp are essential precursors for the synthesis of neurotransmitters–dopamine, epinephrine, norepinephrine and serotonin. Dopaminergic projections in the prefrontal cortex are indispensable to a wide range of cognitive functions, including memory, inhibitory control and cognitive flexibility, which in some PKU patients are impaired [3].

Dietary treatment in PKU, which remains the basic form of therapy for most of patients, requires following a restrictive low-Phe diet. This diet should by optimally introduced in the first days of life which makes it possible to minimalize the neurotoxic effects of a chronic HPA state. The diet is based on a major reduction in natural protein intake, supplementation of protein substitutes that contain Phe-free L-amino acids and the addition of low-protein products as the primary source of energy [4].

Alazami syndrome [OMIM #615071] is an autosomal recessive developmental disorder, manifesting itself in intellectual disability, short stature and distinctive dysmorphic features [5]. It was first described in 2012 by Alazami et al. among a consanguineous Saudi Arabian family [6]. To date, only about 20 affected individuals have been reported worldwide. The underlying molecular mechanism of this condition is linked to a LARP7 gene mutation that is responsible for the stability and functioning of RNA molecules, transcription termination and regulation of translation [7,8]. Most patients present with moderate to severe intellectual disability. Speech development is especially affected [9]. In the first years of a child’s life, failure to thrive and short stature are observed. Dysmorphic features include a triangular face with malar hypoplasia, deep-set eyes with narrow palpebral fissures, sparse eyebrows, wide nasal bridge, short philtrum, full lips and widely spaced teeth [10]. Occasionally, some common features such as low-set ears, prominent forehead, high palate and micrognathia are described [10,11]. In most cases disproportionate mild microcephaly compared with severe short stature is observed, too [10]. Additionally, congenital heart defects and hypospadias have been reported [5,11]. Patients may present with a variety of behavioural disorders, such as excessive agitation, aggressiveness, self-stimulation and autistic features. Cases with friendly and affectionate disposition have also been reported [7]. Brain imaging usually shows no abnormalities, although few patients were diagnosed with ventricular dilatation, hypoplasia of corpus callosum and septum pellucidum [7,9]. In patients with Alazami syndrome, the importance of early intervention, including occupational therapy and intensive speech therapy is emphasized. Timely support allows patients to achieve more efficient motor development and improves their language skills [9].

We present a case of a boy with a classical form of PKU, dysmorphic features and developmental delay, regardless of early implementation of a low-Phe diet and good biochemical control. After many attempts to establish a genetic cause of this unusual phenotype, the boy was finally diagnosed at the age of 16 with Alazami syndrome using whole exome sequencing (WES). We emphasize the need for life-long systematic follow-up in PKU patients. We also draw attention to the problem of the diagnostic odyssey and the usefulness of WES for identifying rare diseases. The following case details some unusual, not previously described, phenotypic presentations of Alazami syndrome.

## 2. A Case Report

**Figure 1:**
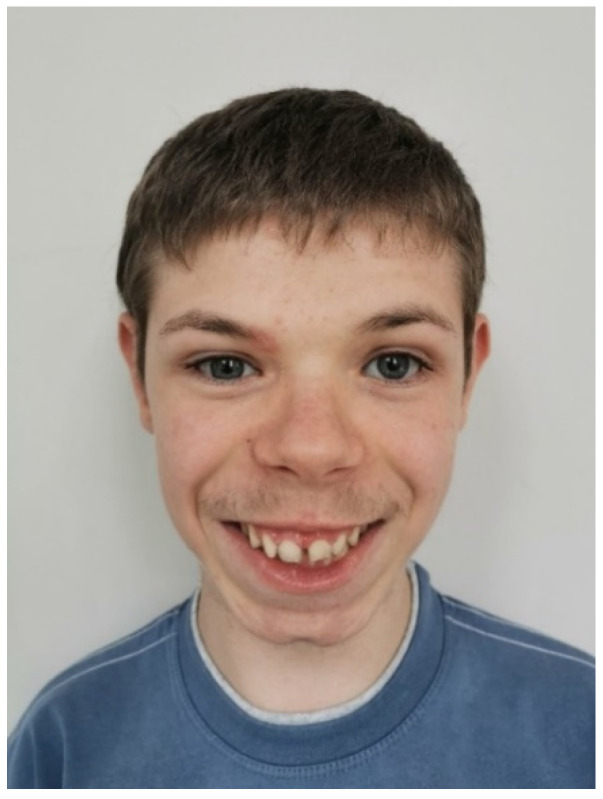
A 16-year-old boy with phenylketonuria and Alazami syndrome (the photo published with consent of the patient’s parents).

**Figure 2:**
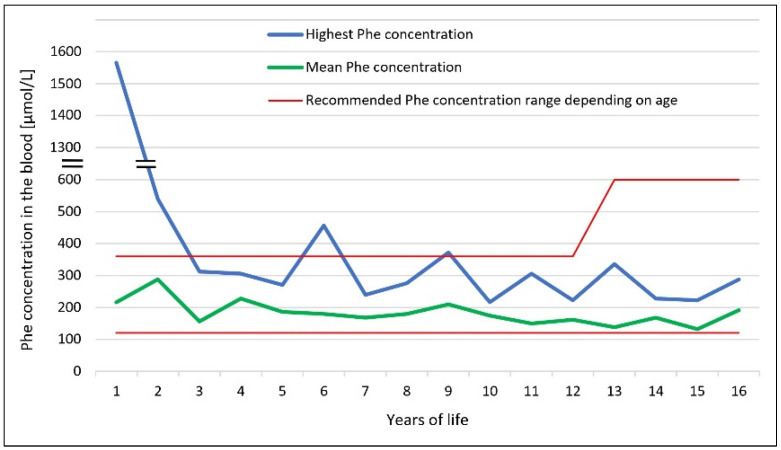
The highest and mean annual blood phenylalanine (Phe) concentrations in a boy suffering from phenylketonuria and Alazami syndrome.

We present a case of a 16-year-old boy with a classical form of PKU and Alazami syndrome. The patient was born at 41 weeks of gestation from an uncomplicated pregnancy via spontaneous vaginal delivery. His birth weight was 2400 g, length 49 cm, Apgar score of 8 points. The boy’s parents were nonconsanguineous and the family genetic history was negative. Discrete nonspecific dysmorphic features were observed after the boy’s delivery. A dried blood spot (DBS) for new-born screening was taken on the 3rd day of life and a HPA positive result (Phe 633 μmol/L, Normal < 120 µmol/L) was obtained on the 9th day of life. On the 11th day of life, the infant was admitted to the Department of Paediatrics. Laboratory tests with Phe concentration amounting to 1564 μmol/L confirmed classical PKU diagnosis. To rule out maternal PKU syndrome in regard to discrete dysmorphic features presented by the child, the mother’s Phe level was also checked and turned out to be within the normal range. In the differential diagnosis of persistent HPA, tetrahydrobiopterin (BH4) deficiency was excluded by determining normal dihydropteridine reductase (DHPR) activity in DBS (5.5 nmol/min/disc) and normal pterin pattern in the urine (neopterin 1657 mmol/L, biopterin 1473 mmol/L). The BH4 loading test (20 mg/kg/dose) was positive, showing a 63% reduction in Phe concentration. A pathogenic variant (missense mutation in exon 12: c.1222C>T) and a likely pathogenic variant (missense mutation in exon 6: c.544G>A) in PAH gene were detected in molecular tests. A low-Phe diet was implemented on the 13th day of the new-born’s life and target blood Phe values (120–360 μmol/L) were quickly reached. The child’s PKU was considered BH4-responsive but sapropterin dihydrochloride is still not available to Polish patients with HPA caused by PAH deficiency responsive to BH4. Despite good metabolic control, the boy’s psychomotor development was delayed in the following months of his life. He started to sit at 9–10 months of age, walk at 15 months and was not able to speak at the age of 2 years. Dysmorphic features also became more prominent: microcephaly, triangular craniofacial shape, large and high-set ears, wide forehead, nasal base, long philtrum, large lower lip and widely spaced teeth (Figure 1). Body weight and height deficiency were observed. The boy was hyperactive, had difficulties with concentration and demonstrated stereotypical behaviour, but at the same time he presented with a cheerful and sociable disposition. He was provided with psychological counselling and an early intervention program was implemented. In laboratory tests, no significant deviations from the norm were found; concentration of ammonia, phosphocreatine kinase, lactate, DBS acylcarnitine profile and organic acids in urine were all in the normal range. Magnetic resonance imaging (MRI) of the head and ultrasound of the heart and abdomen showed no abnormalities. In the following years of life, the child managed to learn basic vocabulary, but the speech was very slurred and behavioural disturbances persisted. The boy’s intelligence measured by the Stanford-Binet 5 Intelligence Scale at the age of 15 was within the lower range of moderate intellectual disability (IQ-36). The clinical symptoms presented in the child did not correlate with Phe concentrations, which most of the time remained within the reference limits (mean annual Phe concentrations ranged from 138 μmol/L to 288 μmol/L) (Figure 2). Initially, molecular testing excluded fragile X syndrome and Williams syndrome, while karyotype and microarray tests (aCGH) showed normal results. Finally, whole exome sequencing was performed (WES; Centogene, Rostock, Germany) and two heterozygous, likely pathogenic, variants in the LARP7 gene were detected (splicing mutation in intron 14 NM_001267039.1: c.1690-1_1692del and frameshift mutation in exon 9: c.855dup). The result was confirmed by Sanger sequencing. Apart from the above variants in LARP7 and PAH genes WES came out with no other clinically relevant findings. In addition to classical PKU, Alazami syndrome was diagnosed based on the characteristic phenotype and the presence of the two variants in LARP7 gene in trans, thereby finally ending the boy’s diagnostic odyssey.

## 3. Discussion

Since the implementation of population new-born screening, PKU can be detected as early as on the third day of life. Early initiation of dietary treatment, ideally in the first 10 days of life, enables achieving normal intellectual development. According to Smith et al. (1990), each four-week delay in diet introduction results in a decrease in intelligence quotient (IQ) by about 4 points [12]. Unfortunately, even in some early diagnosed patients, neurocognitive abilities become impaired. Apart from a lowering IQ, they often involve abnormal higher-order cognitive functioning. These include planning, organization, cognitive flexibility, working memory and inhibitory control [13]. Untreated or poorly controlled PKU leads to significant disorders of psychomotor development and irreversible intellectual disability. Initially, the psychological and psychiatric symptoms presented by our patient raised the suspicion of poor PKU metabolic control. In a situation like this, it is crucial to assess blood Phe levels not only in current measurements, but also throughout a patient’s life. According to the European Guidelines for Diagnosis and Treatment of Phenylketonuria published in 2017, target blood Phe levels in children up to 12 years of age should stay within the values of 120–360 μmol/L, while in patients over 12 years of age in the range of 120–600 μmol/L [2]. After implementing a low-Phe diet, the highest Phe concentration in our patient during his neonatal period was 540 μmol/L. In the following months and years of life, the mean annual Phe concentrations ranged from 138 μmol/L to 288 μmol/L. These values suggest that the dietary recommendations were followed and HPA could have been excluded as the cause of intellectual disability and behavioural disorders.

In a patient with PKU, apart from regular metabolic control, a physician’s attention should be drawn to clinical symptoms unusual for this disease. This problem was highlighted by MacDonald et al. (2015) who reported 30 patients with PKU and a coexistent rare disorder [14]. In the presented case, dysmorphic features prompted the physician to conduct a genetic investigation. If WES testing had not been performed, the final diagnosis of the ultra-rare Alazami syndrome would not be arrived at. The patient presented some symptoms typical for this disease (intellectual disability, short stature, microcephaly, triangular craniofacial shape, wide nose, widely spaced teeth), as well as large high-set ears and a long philtrum; the two features that have not been described so far. Unlike most Alazami patients with aggressive or autistic behaviour, our patient showed a friendly and happy disposition. Thanks to early intervention, the boy mastered basic vocabulary and became independent in most important daily activities.

## 4. Conclusions

In this publication we presented a case of a patient with a classical form of PKU who was additionally diagnosed with Alazami syndrome. This ultra-rare disease is characterized by moderate to severe intellectual disability, severe growth deficiency and a set of dysmorphic features. We described two unusual symptoms for this syndrome: large high-set ears and a long philtrum. It was thanks to WES testing that the boy’s final diagnosis was reached. This revolutionary molecular analysis of monogenic diseases makes it possible to evaluate the entire DNA coding sequence and significantly changes our approach to the diagnosis of rare diseases that are difficult to identify. Whole exome sequencing offers the possibility of replacing long-term, multi-directional and costly investigation of rare diseases with a single test. Our publication highlights the need for life-long systematic follow-up of PKU patients, as well as the risk of abnormal intellectual development and neuropsychological deficits caused by chronic HPA. In conclusion, it should be underlined that the presence of one monogenic disease does not exclude the coexistence of another one. If a patient has atypical symptoms, further research becomes necessary to determine a final diagnosis.
